# Enteric dysfunction and other factors associated with attained size at 5 years: MAL-ED birth cohort study findings

**DOI:** 10.1093/ajcn/nqz004

**Published:** 2019-05-25

**Authors:** Stephanie A Richard, Benjamin J J McCormick, Laura E Murray-Kolb, Gwyneth O Lee, Jessica C Seidman, Mustafa Mahfuz, Tahmeed Ahmed, Richard L Guerrant, William A Petri, Elizabeth T Rogawski, Eric Houpt, Gagandeep Kang, Estomih Mduma, Margaret N Kosek, Aldo A M Lima, Sanjaya K Shrestha, Ram K Chandyo, Zulfiqar Bhutta, Pascal Bessong, Laura E Caulfield, MAL-ED Network Investigators

**Affiliations:** 1 Fogarty International Center/NIH, Bethesda, MD; 2 The Pennsylvania State University, University Park, PA; 3 The Johns Hopkins University, Baltimore, MD; 4 International Centre for Diarrhoeal Disease Research, Bangladesh (icddr,b), Dhaka, Bangladesh; 5 University of Virginia, School of Medicine, Charlottesville, VA; 6 Christian Medical College, Division of Gastrointestinal Sciences, Vellore, Tamil Nadu, India; 7 Haydom Lutheran Hospital, Haydom, Manyara, Tanzania; 8 Universidade Federal do Ceará, INCT—Instituto de Biomedicina do Semiárido Brasileiro, Fortaleza, Brazil; 9 Walter Reed Armed Forces Research Institute of Medical Sciences (AFRIMS) Research Unit (WARUN), Kathmandu, Nepal; 10 Institute of Medicine, Tribhuvan University, Kathmandu, Nepal; 11 Aga Khan University, Centre of Excellence in Women and Child Health, Karachi, Pakistan; 12 University of Venda, Thohoyandou, South Africa

**Keywords:** growth, enteric dysfunction, iron, inflammation, permeability

## Abstract

**Background:**

Poor growth in early childhood has been associated with increased risk of mortality and morbidity, as well as long-term deficits in cognitive development and economic productivity.

**Objectives:**

Data from the MAL-ED cohort study were used to identify factors in the first 2 y of life that are associated with height-for-age, weight-for-age, and body mass index *z*-scores (HAZ, WAZ, BMIZ) at 5 y of age.

**Methods:**

A total of 1017 children were followed from near birth until 5 y of age at sites in Bangladesh, Brazil, India, Nepal, Peru, South Africa, and Tanzania. Data were collected on their growth, environmental enteric dysfunction (EED), micronutrient status, enteric pathogen burden, illness prevalence, dietary intake, and various other socio-economic and environmental factors.

**Results:**

EED biomarkers were related to size at 5 y. Mean lactulose:mannitol *z*-scores during the first 2 y of life were negatively associated with all of the growth measures (HAZ: −0.11 [95% CI: −0.19, −0.03]; WAZ: −0.16 [95% CI: −0.26, −0.06]; BMIZ: −0.11 [95% CI: −0.23, 0.0]). Myeloperoxidase was negatively associated with weight (WAZ: −0.52 [95% CI: −0.78, −0.26] and BMIZ: −0.56 [95% CI: −0.86, −0.26]); whereas α-1-antitrypsin had a negative association with HAZ (−0.28 [95% CI: −0.52, −0.04]). Transferrin receptor was positively related to HAZ (0.18 [95% CI: 0.06, 0.30]) and WAZ (0.21 [95% CI: 0.07, 0.35]). Hemoglobin was positively related to HAZ (0.06 [95% CI: 0.00, 0.12]), and ferritin was negatively related to HAZ (−0.08 [95% CI: −0.12, −0.04]). Bacterial density in stool was negatively associated with HAZ (−0.04 [95% CI: −0.08, 0.00]), but illness symptoms did not have any effect on size at 5 y.

**Conclusions:**

EED markers, bacterial density, and iron markers are associated with growth at 5 y of age. Interventions to reduce bacterial burden and EED may improve long-term growth in low-income settings.

## Introduction

Attained size at 5 y of age represents the summation of factors influencing growth in utero and early childhood (1). Small size at birth, inadequate dietary intake, and morbidity lead to growth faltering during the first 2 y of life, and this faltering may not be recovered over the preschool period (1–4). Indeed, persistent stunting is a worldwide problem profoundly affecting functioning throughout life (5). More recent longitudinal studies from low- and middle-income countries (LMICs) involving follow-up beyond 2 y have identified socio-economic status, parental education, and maternal height as factors that influence longer-term outcomes for children living in poverty today (6).

In 1991, Lunn et al. (7) reported that intestinal permeability and mucosal injury contributed to growth faltering in Gambian infants. Over time, this area of research has expanded as investigators have considered how the impacts of repeated enteric infections and poor diet impact the functional capacities of the gut, and the role of what is now called environmental enteric dysfunction (EED) in the etiology of stunting (8, 9). There are multiple pathways through which EED may affect growth faltering and stunting (9, 10), including increased intestinal and systemic inflammation, and increased intestinal permeability, which can result in reduced absorptive capacity and altered nutrient status. Few studies have so far evaluated whether early markers of gut dysfunction are related to growth over the long term (11).

The "Etiology, Risk Factors, and Interactions of Enteric Infections and Malnutrition and Consequences for Child Health and Development (MAL-ED)" birth cohort study aimed to evaluate the relations among EED, enteric pathogens, inadequate dietary intake, growth, and cognitive development of children in LMICs (12). Infants were enrolled near birth in 8 LMICs and followed to characterize feeding, morbidity, enteropathogen exposure, and EED over the first 2 y of life. Previous papers from this study highlighted subclinical enteropathogen infection and the complementary feeding diet as influential factors affecting growth velocity and the development of stunting; the associations between EED biomarkers and linear growth were detectable but small in 1 analysis (8), and nondetectable or inconsistent in others (13, 14). The EED biomarkers are along the hypothesized pathway between enteropathogen exposure and growth, and prior analyses demonstrated stronger associations with bacterial pathogen exposures (8). We followed up the MAL-ED children with an assessment of attained size at 5 y of age to evaluate the influence of factors in the first 2 y of life on attained stature and size at age 5, with the goals of identifying early life factors with persistent influence on growth outcomes and re-evaluating the effect of EED on growth over the long-term.

## Methods

The MAL-ED study was conducted in Dhaka, Bangladesh; Fortaleza, Brazil; Vellore, India; Bhaktapur, Nepal; Loreto, Peru; Naushero Feroze, Pakistan; Venda, South Africa; and Haydom, Tanzania. Overall, 2145 infants were enrolled, and the initial 24-mo study was implemented from November 2009 until February 2014, with extended follow-up from November 2014 until February 2017. Enrolled infants were less than 17 d old, born singleton with a birth weight >1500 g, without serious illnesses, to a mother at least 16 y of age, and to a family intending to stay in the community for at least 6 mo. The methods for original data collection are published elsewhere (15–18), but relevant details informative to this work are provided below, as well as the methods for follow-up at 5 y of age.

### Ethical approval

Each site obtained ethical approval from their respective institutions, and written consent was obtained from participants for the original study and subsequently for the follow-up.

### Growth measures

Trained field personnel measured the height and weight of the children upon enrollment, monthly during the first 2 y of life, and on their 5th birthday (60 mo ± 1 mo). Previously, we reported problems with the early anthropometric measures from Naushero Feroze, Pakistan, (13), and so we have excluded Naushero Feroze, Pakistan, data from this analysis. We converted observations to height-for-age (HAZ), weight-for-age (WAZ), weight-for-height (WHZ), and body mass index (BMIZ) *z*-scores using the WHO growth standards (19). The primary outcomes were HAZ, WAZ, and BMIZ, with WHZ considered a secondary outcome. Maternal height and weight were measured at 2 mo postpartum.

### Illness and microbiology

During the first 2 y of life, caregivers were visited in their homes twice a week and asked whether the child had been ill or had experienced various symptoms each day since the last visit (15, 20). For these analyses, children had to contribute at least 700 d of illness surveillance in the first 2 y of life. Illnesses (i.e., diarrhea, acute lower respiratory infections, fever, maternal report of nonspecific illness) were summarized as the percentage of days with the illness during the first 2 y of life.

Enteropathogen burden from nondiarrheal stools collected monthly in the first year of life and quarterly in the second year was characterized as the mean number of pathogens per stool sample (16, 21). Children were required to have at least 8 fully tested stool samples in order to be included. Pathogens were also classified as bacterial, viral, or parasitic, and were included as the proportion of nondiarrheal stool samples that were positive for each. Specific pathogens were also tested as the proportion of nondiarrheal stool samples that were positive for each pathogen. Quantitative PCR using TaqMan Array Cards (ThermoFisher, Carlsbad, CA) was used to re-analyze stool samples (22); a sensitivity analysis was performed using the TaqMan data, comparing the results with those derived using traditional microbiologic methods.

### Biomarkers of EED

The lactulose:mannitol (L:M) test is utilized to evaluate intestinal permeability and was administered at 3, 6, 9, and 15 mo (17). Urinary excretion of lactulose (%) and mannitol (%) was determined, as well as their ratio, expressed as standardized L:M *z*-scores (LMZ) (23). Children with at least 3 L:M tests were included in the final data set, and these observations were averaged for each child. We considered both averaged and individual LMZ measures, as well as the individual measures of lactulose and mannitol in the analyses.

Fecal measures of gut inflammation and permeability were characterized in nondiarrheal stool samples collected monthly during the first year of life and quarterly in the second year using myeloperoxidase (MPO), neopterin, and α-1-antitrypsin (AAT) (8). Children with at least 8 assessments were included in the model. A regression for each of the log-transformed fecal biomarkers was used to detrend for age, recent maternally reported fever and breast milk consumption, stool consistency, and the time between collection and sample testing (24). Residuals from these models were averaged for each child.

### Early infant feeding and child diet

Breastfeeding was characterized for each child by the duration of exclusive breastfeeding and the duration of any breastfeeding from twice-weekly interviews during the first 2 y of life. Nonbreast milk food intake was quantified using a 24-h recall monthly implemented by trained personnel in the caregivers’ homes from 9 to 24 mo of age (18). The usual energy, macronutrient, and micronutrient intakes were calculated by averaging at least 11 observations. Nutrient densities were calculated by adjusting nutrient intakes for energy using a residual model having normalized intakes (Box-Cox) and creating a standard normal *z*-score, by site, from the nutrient residuals (25). Overall protein density was found to be representative of a higher-quality diet based on analyses of the correlation structure among individual nutrients.

### Micronutrient status

Venous blood samples were collected at 7, 15, and 24 mo to characterize the status of vitamin A (plasma retinol), zinc (plasma zinc), and iron (plasma ferritin, transferrin receptor [TfR]), as well as systemic inflammation (plasma α-1-acid glycoprotein [AGP]). A finger-prick blood sample was obtained to determine hemoglobin concentration. Concentrations of both TfR and ferritin were adjusted for inflammation using the Biomarkers Reflecting Inflammation and Nutritional Determinants of Anemia (BRINDA) method (26, 27), by age and site, and hemoglobin concentration was adjusted for altitude where appropriate. Children were required to have at least 1 observation, and 92% of children had more than 1 observation, which were averaged. TfR and ferritin concentrations were transformed using a square root function.

### Socio-economic status

At 6, 12, 18, and 24 mo of age, families were asked about household assets and income, type of sanitation, source of drinking-water, hand-washing behaviors, and maternal education. The MAL-ED study developed a socio-economic index referred to as the Water, Assets, Maternal education, and household Income (WAMI) index to provide a cross-site measure of socio-economic status (28). The mean of the WAMI index from 6–24 mo was used to adjust for differences in socio-economic status across children.

### Sample size

Of the 1868 infants enrolled in the study at the 7 sites, 1498 (80%) remained in the study at 24 mo (**Table 1** and **Supplemental Figure 1**). Of these, 1188 (79%) had anthropometric measurements taken at 5 y. The sample size was reduced to 1131 (95% of those in the follow-up cohort) based on the criteria for data completeness (illness, microbiology, and diet), and then finally to 1017 children (86% of those with 5-y anthropometry) based on data completeness for other exposure variables (maternal height, gut inflammation and permeability, and micronutrient status). We compared the children enrolled in the cohort with those who were included in the 5-y analysis. Overall, children included in the analysis had mothers with less education, households with lower WAMI scores, and worse WAZ and weight-for-length *z*-score at enrollment than children who were not included (**Supplemental Table 1**), but these differences were rarely statistically significant at the site level.

**TABLE 1:** Subjects included in the analysis, from enrollment to final number with complete data, *n* (%)^1^

	Southern Asia			Latin America		Sub-Saharan Africa		Total
	BGD	INV	NEB	BRF	PEL	SAV	TZH	
Enrolled	265	251	240	233	303	314	262	1868
Follow-up to 2 y^2^	213 (80)	228 (91)	228 (95)	169 (73)	208 (69)	237 (75)	215 (82)	1498 (80)
Anthropometry at 5 y^3^	193 (91)	213 (93)	128 (56)	131 (78)	164 (79)	183 (77)	176 (82)	1188 (79)
Complete illness, pathogen history, and complementary diet data during early childhood^4^	193 (100)	212 (99)	127 (99)	111 (85)	154 (94)	164 (90)	170 (97)	1131 (95)
Complete data on other variables^5^	186 (96)	207 (97)	122 (95)	99 (76)	145 (88)	132 (72)	126 (71)	1017 (86)

1 Sites: BGD: Bangladesh—Dhaka; INV: India—Vellore; NEB: Nepal—Bhaktapur; BRF: Brazil—Fortaleza; PEL: Peru—Loreto; SAV: South Africa—Venda; TZH: Tanzania—Haydom.

2 Children with follow-up to 2 y included in analyses of 2-y growth outcomes (percentage of those enrolled).

3 Children with height and weight at 5 y of age (percentage of those still in study at 2 y). In SAV (4) and NEB (99), children were older than 62 mo when funding (SAV) or ethical clearance (NEB) was obtained.

4 Children with at least 700 d of illness surveillance, 11 measures of dietary intake from 9 to 24 mo, 8 stool samples to detect enteropathogens and fecal biomarkers of gut inflammation (percentage of those in the follow-up study).

5 Children with data on maternal height, micronutrient status and L:M test results (percentage of those in the follow-up study).

### Statistical analyses

Multivariable linear regression was used to evaluate exposures in the first 2 y of life with 5-y growth outcomes in 4 different models: HAZ, WAZ, BMIZ, and WHZ. Candidate variables were evaluated that represented the domains of illness and pathogen burden, breastfeeding, dietary intake from nonbreast milk foods, EED, micronutrient status, and child and household characteristics (**Supplemental Table 2**). The final set of variables reflected explicit evaluation of our research questions, biological rationale, and stepwise selection (optimizing the Akaike Information Criterion). Sites were included as dummy variables. Because we were explicitly interested in evaluating whether aspects of EED mediate the relations between diet and/or pathogen burden and growth, models were run separately with either the EED variables or diet and pathogen burden variables, and then a combined (final) model. Univariate model results are included in **Supplemental Table 3**. Analyses were performed with R 3.4.3 (Foundation for Statistical Computing) using the lm and ggplot packages.

## Results

The mean HAZ of the children declined from enrollment to 5 y of age at 6 of the 7 sites (**Table 2**). In Fortaleza, Brazil, the mean length-for-age *z*-score was −0.8 at enrollment, but increased to −0.2 HAZ at 5 y of age. With the exception of Venda, South Africa, and Haydom, Tanzania, the mean WHZ increased slightly over time. Selected measures of the early child environment are provided for each site in Table 2.

**TABLE 2:** Mean attained size at 5 y of age and selected characteristics measured during the first 2 y of life by site (mean [SD] unless otherwise specified)^1^

	Southern Asia			Latin America		Sub-Saharan Africa	
	BGD	INV	NEB	BRF	PEL	SAV	TZH
*n*	186	207	122	99	145	132	126
Outcomes							
HAZ at 60 mo	−1.6 (0.9)	−1.5 (0.9)	−1.3 (0.9)	−0.2 (1.0)	−1.3 (0.8)	−0.9 (1.0)	−1.9 (0.9)
WAZ at 60 mo	−1.5 (1.0)	−1.6 (0.9)	−1.0 (0.8)	0.3 (1.4)	−0.5 (1.0)	−0.8 (1.1)	−1.4 (0.8)
BMIZ at 60 mo	−0.8 (1.0)	−0.9 (0.9)	−0.3 (0.8)	0.6 (1.5)	0.5 (1.0)	−0.4 (1.4)	−0.3 (1.0)
WHZ at 60 mo	−0.9 (1.0)	−1.0 (1.0)	−0.3 (0.9)	0.6 (1.5)	0.4 (1.1)	−0.4 (1.4)	−0.4 (1.0)
Contributing factors							
LAZ at enrollment	−1.0 (1.0)	−1.0 (1.1)	−0.7 (1.0)	−0.8 (1.2)	−1.0 (0.9)	−0.8 (1.0)	−1.0 (1.2)
WAZ at enrollment	−1.3 (0.9)	−1.3 (1.1)	−0.9 (1.0)	−0.1 (1.1)	−0.7 (0.9)	−0.4 (1.0)	−0.2 (1.0)
WLZ at enrollment	−1.1 (1.0)	−1.2 (1.1)	−0.9 (1.1)	0.5 (1.3)	−0.0 (1.0)	0.1 (1.3)	0.7 (1.2)
WAMI	0.5 (0.1)	0.5 (0.1)	0.7 (0.1)	0.8 (0.1)	0.5 (0.1)	0.8 (0.1)	0.2 (0.1)
Males, %	91 (49)	113 (55)	66 (54)	41 (41)	66 (46)	63 (48)	65 (52)
Maternal height, cm	149 (5.0)	151 (5.0)	150 (4.9)	155 (6.7)	150 (5.4)	159 (6.6)	156 (5.9)
Days of maternally reported illness per year	193 (57)	152 (65)	61 (39)	19 (15)	21 (13)	16 (12)	38 (21)
Energy from complementary feeding, kcal/d	357 (130)	751 (220)	436 (168)	987 (198)	742 (170)	878 (185)	1009 (182)
Protein intake from complementary feeding, g/d	10 (4)	22 (8)	12 (5)	41 (9)	20 (5)	28 (6)	29 (6)
Hemoglobin, g/dL	11.4 (1.2)	10.9 (0.9)	10.4 (1.0)	11.4 (1.3)	11.1 (0.9)	11.0 (1.1)	11.1 (1.2)
Transferrin receptor, mg/L	6.6 (2.8)	4.5 (2.0)	8.8 (2.8)	9.7 (2.4)	7.3 (2.0)	4.0 (2.0)	4.6 (1.9)
Ferritin, µg/L	15.6 (9.9)	13.9 (11.5)	13.0 (10.4)	19.8 (8.8)	23.5 (18.0)	22.9 (16.1)	16.4 (11.1)
Pathogen density (number of pathogens per monthly stool sample)	1.1 (0.3)	1.1 (0.4)	0.8 (0.3)	1.2 (0.4)	0.9 (0.4)	0.7 (0.3)	1.4 (0.4)
Bacterial density (proportion of stool samples with bacteria)	0.6 (0.2)	0.6 (0.2)	0.5 (0.2)	0.6 (0.2)	0.4 (0.2)	0.4 (0.1)	0.7 (0.2)
Lactulose:mannitol *z*-score	0.3 (0.5)	0.5 (0.5)	0.0 (0.5)	−0.0 (0.6)	0.6 (0.4)	0.6 (0.9)	0.4 (0.7)
α-1-Antitrypsin, ng/mL	0.4 (0.1)	0.4 (0.1)	0.4 (0.1)	0.3 (0.1)	0.4 (0.2)	0.2 (0.1)	0.3 (0.2)
Myeloperoxidase, ng/mol	4380 (1834)	7866 (3959)	4166 (1880)	3008 (2394)	7950 (3878)	4614 (1750)	5511 (2520)
Neopterin, nmol/L	1060 (471)	1772 (653)	1598 (459)	1596 (541)	2400 (858)	3859 (1086)	875 (500)

1 Sites: BGD: Bangladesh—Dhaka; INV: India—Vellore; NEB: Nepal—Bhaktapur; BRF: Brazil—Fortaleza; PEL: Peru—Loreto; SAV: South Africa—Venda; TZH: Tanzania—Haydom.

### HAZ outcome

**Figure 1:**
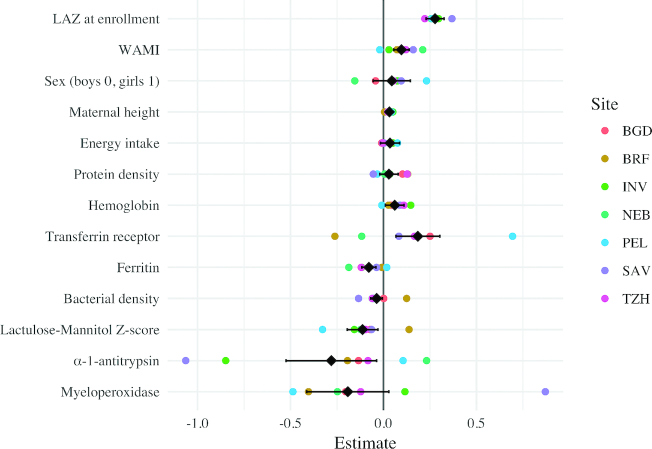
Multivariable linear regression models were used to identify factors in early childhood that were associated with height-for-age*z*-scores (HAZ) at 5 y of age. The overall model (in black) includes site as a fixed effect. Site-specific models were run, and the coefficients from those models are plotted in color. Sites: BGD: Bangladesh—Dhaka; INV: India—Vellore; NEB: Nepal—Bhaktapur; BRF: Brazil—Fortaleza; PEL: Peru—Loreto; SAV: South Africa—Venda; TZH: Tanzania—Haydom.

Child, household, and maternal characteristics, as well as measures of iron status, bacterial density, and EED, were associated with HAZ at 5 y of age (**Table 3**, **Figure 1**, **Supplemental Tables 4–7**). Socio-economic status (WAMI), length-for-age *z*-score at enrollment, and maternal height were each positively associated with HAZ at 5 y of age. Sex, energy intake, and protein density did not have statistically significant associations with HAZ at 5 y of age. Mean hemoglobin and TfR concentrations were each positively associated with HAZ at 5 y of age (0.06 [95% CI: 0.00, 0.12] and 0.18 [95% CI: 0.06, 0.30], respectively), whereas mean ferritin concentration was negatively associated (−0.08 [95% CI: −0.12, −0.04]). Bacterial density was negatively associated with 5-y HAZ (−0.04 HAZ per 10% increase in bacteria positive samples [95% CI: −0.08, 0.00]), as were mean LMZ and mean detrended log fecal AAT concentration in the first 2 y of life (−0.11 [95% CI: −0.19, −0.03] and −0.28 [95% CI: −0.52, −0.04], respectively). In separate models, individual measures of LMZ showed no trend in association with HAZ (Supplemental Table 5); further, although not statistically significant, lactulose excretion was negatively associated, whereas mannitol excretion was positively associated with HAZ (Supplemental Tables 6 and 7). Adding EED variables reduced the effect size and statistical significance of the energy intake variable and MPO, but did not have a meaningful impact on the bacterial density interpretation (Supplemental Table 4). The bacterial density measured by the TaqMan assay was also associated with lower HAZ at 5 y of age (−0.06 [95% CI: −0.1, −0.02]) (**Supplemental Figure 2**).

**TABLE 3:** Multivariable linear regression model results considering early childhood factors associated with growth at 5 y of age

	HAZ^1^	WAZ^1^	BMIZ^1^	WHZ^1^
LAZ at enrollment	0.28 (0.02)****	—	0.06 (0.03)	—
WAZ at enrollment	—	0.27 (0.03)***	—	—
WLZ at enrollment	—	—	—	0.19 (0.03)***
WAMI	0.10 (0.02)***	0.11 (0.03)***	0.07 (0.03)*	0.08 (0.03)*
Sex (boys 0, girls 1)	0.04 (0.05)	−0.05 (0.06)	−0.18 (0.07)*	−0.05 (0.07)
Maternal height, cm	0.03 (0.00)***	0.03 (0.01)***	0.00 (0.01)	0.01 (0.01)
Energy intake^2^	0.03 (0.03)	0.05 (0.03)	0.03 (0.04)	0.04 (0.04)
Protein density^2^	0.03 (0.03)	0.00 (0.03)	−0.03 (0.03)	−0.03 (0.03)
Hemoglobin	0.06 (0.03)*	0.02 (0.03)	−0.01 (0.03)	−0.03 (0.04)
Transferrin receptor^3^	0.18 (0.06)**	0.21 (0.07)**	0.14 (0.08)	0.13 (0.08)
Ferritin^3^	−0.08 (0.02)***	−0.05 (0.02)*	0.02 (0.03)	0.01 (0.03)
Bacterial density	−0.04 (0.02)*	−0.02 (0.02)	0.01 (0.02)	0.01 (0.02)
Lactulose:mannitol *z*-score	−0.11 (0.04)**	−0.16 (0.05)***	−0.11 (0.06)*	−0.14 (0.06)*
α-1-Antitrypsin^4^	−0.28 (0.12)*	−0.20 (0.15)	0.06 (0.17)	−0.00 (0.17)
Myeloperoxidase^4^	−0.19 (0.11)	−0.52 (0.13)***	−0.56 (0.15)***	−0.60 (0.16)***
Adj. *R*^2^	0.41	0.38	0.23	0.26
Number of observations	1017	1017	1017	1015

* *P* < 0.05, **P< 0.01, ^***^P< 0.001.

1 Height-for-age (HAZ), weight-for-age (WAZ), body mass index *z*-score (BMIZ), and weight-for-height *z*-score (WHZ). Values are coefficient (SE). The models include site as a fixed effect (results not shown).

2 Variables have been standardized; the beta estimates represent the difference in 1 SD change in the variable.

3 Variables have been adjusted for inflammation and normalized by taking the square root.

4 Mean log concentration (detrended).

### WAZ outcome

Similar to the HAZ model, child, household, and maternal characteristics, as well as markers of iron status and EED were associated with WAZ at 5 y of age (Table 3, **Supplemental Figure 3**, and **Supplemental Table 8**). Socio-economic status (WAMI), WAZ at enrollment, and maternal height were all positively associated with WAZ at 5 y of age. Mean TfR concentration in the first 2 y of life was positively associated with WAZ (0.21 [95% CI: 0.07, 0.35]), and mean ferritin was negatively associated with WAZ (−0.05 [95% CI: −0.09, −0.01]). Mean LMZ in the first 2 y of life was again negatively associated with WAZ at 5 y of age (−0.16 [95% CI: −0.26, −0.06]), but the relation with AAT was not statistically significant. However, mean MPO concentration was negatively associated with WAZ (−0.52 [95% CI: −0.78, −0.26]). EED variables reduced the effect size of diet and bacterial density variables, and, in the case of energy intake, statistical significance. Sex, energy intake, protein density, hemoglobin, bacterial density, and AAT were not found to be significantly associated with WAZ at 5 y of age in the final model.

### BMIZ outcome

Overall, BMIZ at 5 y of age was associated with socio-economic status (WAMI) (0.07 [95% CI: 0.01, 0.13]), sex of the child (−0.18 [95% CI: −0.32, −0.04]), LMZ (−0.11 [95% CI: −0.23, 0.0]), and mean MPO concentration (−0.56 [95% CI: −0.86, −0.26]) (Table 3, **Supplemental Figure 4**, and **Supplemental Table 9**). In contrast to the results of the HAZ model, none of the iron status biomarkers were significantly related to BMIZ at 5 y of age. The association between maternal height and BMIZ was not significant. The BMIZ model was the only model in which sex was a significant factor—girls had lower BMIZ scores than boys. Energy intake, protein density, AAT, and bacterial density during the first 2 y of life were not significantly associated with BMIZ at 5 y of age.

Also presented in Table 3 are the results for WHZ; as shown for HAZ and WAZ, the mean LMZ and mean MPO concentration were each negatively associated with WHZ, but as shown for BMIZ, no associations with iron status measures were detected.

## Discussion

Our findings provide new evidence that EED in early childhood is associated with reduced stature, weight, weight-for-height, and BMI at 5 y of age. Intestinal permeability, assessed by LMZ, was negatively associated with each of these measures, adjusting for multiple covariates and potentially confounding factors. The mean concentrations of 2 fecal biomarkers were also negatively related to growth, the mean AAT concentration with HAZ, and the mean MPO concentration with each of the weight-associated outcomes. Importantly, and related to these measures (8, 24), a child's exposure to bacterial enteropathogens in nondiarrheal stools was associated negatively with HAZ alone. Bacterial density was found to have a negative effect on growth outcomes at 5 y, whereas viral and parasitic pathogen densities did not. Overall, these results indicate that bacterial burden and EED during the first 2 y of life can negatively affect growth to age 5 y. Harper et al. (29) recently conducted a review of EED and stunting, and concluded that EED pathways involving intestinal and systemic inflammation had more consistent support than did pathways involving permeability and mucosal damage, but our results provide empirical support for both pathways. Describing these relations is important because, although diarrhea is an important cause of morbidity and mortality in low-income countries (30), studies have noted that diarrheal incidence and mortality have declined more strongly over time than has the prevalence of stunting (30–33). Our results indicate that pervasive but clinically overlooked EED and pathogen burdens have long-term consequences for child growth and may provide some explanation for these global trends.

Previously, we used linear splines to explore the relations between many of the variables included here and growth up to 24 mo of age (13). In that analysis, we found that pathogen density was negatively associated with HAZ at 24 mo of age, and this corresponds well to our more specific finding in the current manuscript that bacterial density in the first 2 y of life is associated with lower HAZ at 60 mo of age. Conversely, although we found that higher-quality complementary feeding was associated with linear growth in the first 2 y of life, this finding was not supported in the current manuscript. Although both mean energy intake and protein density in the first 2 y of life were positively associated with 60-mo HAZ in the univariate models (Supplemental Table 3), inclusion of other variables in the model resulted in positive, but not statistically significant, findings. Potential factors associated with the discrepancy between the 2- and 5-y results include that the complementary feeding practices during the first 2 y of life may not necessarily represent later dietary intake, or that other factors potentially play a larger role in height attainment in the 3–5-y period. Consistent with our analysis of growth velocity in the first 2 y of life (13), we found no evidence of a long-term association between illness symptoms (diarrhea, respiratory infections, etc.) in the first 2 y of life and size at 5 y of age. Although very different methods were used in the initial 2-y analysis, many of the findings were similar when looking at the 5-y growth outcomes, indicating that the factors associated with growth at 2 y of age either have a long-term effect on the growth of children or represent factors that continue to diminish the growth of these children in these environments.

We found that the mean hemoglobin and TfR concentrations (at 7, 15, and 24 mo) were each positively related, whereas mean ferritin concentrations were negatively related, to HAZ at age 5; TfR was uniquely and positively also associated with WAZ. Interpreting these findings is difficult because the measures are interrelated, and because they are affected by multiple physiologic processes, involving rapid growth, erythropoiesis, and developmental changes in the regulation of iron metabolism. Iron stores (e.g., as indicated by higher plasma ferritin) must be adequate for erythropoiesis (34), and ferritin and hemoglobin are positively correlated. TfR is generally elevated with iron-deficient erythropoiesis, but it is also elevated with cellular proliferation (35) and, here, may be more reflective of variability in erythropoiesis across children. The findings that anemia and iron deficiency are not associated with growth outcomes support this interpretation. Both ferritin and TfR concentrations were elevated in response to inflammation, but we adjusted the concentrations using AGP before analyses (26, 27). Our small negative associations between mean ferritin concentration and HAZ at 5 y may reflect incomplete adjustment for systemic inflammation, or inflammation-induced binding of hepcidin to ferroportin, which would trap iron within cells, rendering it unavailable to support the rapid erythropoiesis characteristic of early childhood (36). Research is needed to delineate how these processes lead to detectable differences in growth.

Strengths of the study include the harmonized protocol across 7 sites and intensity of the follow-up with detailed assessments of morbidity, enteropathogen exposure, and diet, as well as measures of multiple pathways through which EED would affect growth outcomes. The results of the analyses were fairly consistent across sites (Supplemental Figures 2–4). We conducted sensitivity analyses to better understand our LMZ and fecal biomarkers and demonstrated that our results regarding bacterial density are robust across assay methodologies. Our ability to follow up these children is another strength, limited by time delays to obtain ethical clearance once funding was secured at some sites; however, the loss of children over the 5-y period was a limitation, mostly due to movement out of the catchment area. Another limitation of the study was the break in funding for follow-up, which led to inconsistent collection of data after 2 y of age among the sites, which limited our ability to include data from 24–57 mo in a multisite model.

In summary, bacterial pathogen exposure in early childhood and longitudinal measures of EED are associated with reduced growth outcomes at age 5. Environmental interventions to reduce bacterial burdens and hence EED may have implications for long-term growth. Exposure to enteropathogens and EED (primarily through inflammation) in early childhood affect risk of obesity and cardiometabolic risk factors beyond the preschool period (11, 37–39), which underscore the need for prevention and management in early childhood. Further research is needed to understand erythropoiesis and regulation of iron homeostasis during infancy in settings with EED and low intakes of iron to fully interpret our findings. Further follow-up of these children may provide additional evidence toward understanding the importance of early life events that affect the functioning of the gut on outcomes in later life.

## Supplementary Material

nqz004_Supplemental_File

## Notes

The Etiology, Risk Factors and Interactions of Enteric Infections and Malnutrition and the Consequences for Child Health and Development Project (MAL-ED) is carried out as a collaborative project supported by the Bill & Melinda Gates Foundation, the Foundation for the NIH, and the NIH/Fogarty International Center. This work was also supported by the Fogarty International Center, NIH (D43-TW009359 to ETR).

Supplemental Figures 1–4 and Supplemental Tables 1–9 are available from the “Supplementary data” link in the online posting of the article and from the same link in the online table of contents at https://academic.oup.com/ajcn/.

Abbreviations used: AAT, α-1-antitrypsin; BMIZ, body mass index *z*-score; EED, environmental enteric dysfunction; HAZ, height-for-age *z*-score; L:M, lactulose:mannitol; LMZ, lactulose:mannitol *z*-score; LMIC, low- and middle-income country; MAL-ED, Etiology, Risk Factors, and Interactions of Enteric Infections and Malnutrition and Consequences for Child Health and Development; MPO, myeloperoxidase; TfR, transferrin receptor; WAMI, Water, Assets, Maternal education, and household Income; WAZ, weight-for-age *z*-score; WHZ, weight-for-height *z*-score.

## References

[1] McDonald CM, Thorne-Lyman A The importance of the first 1,000 days: An epidemiological perspective. In: The biology of the first 1000 days. Boca Raton, FL: CRC Press, Taylor & Francis Group; 2017 p. 3–13.

[2] Golden MH Is complete catch-up possible for stunted malnourished children?. Eur J Clin Nutr. 1994;48 Suppl 1:S58–70.; discussion S71. 8005092

[3] Martorell R, Khan LK, Schroeder DG Reversibility of stunting: Epidemiological findings in children from developing countries. Eur J Clin Nutr. 1994;48 Suppl 1:S45–57. 8005090

[4] Moore SR, Lima AA, Conaway MR, Schorling JB, Soares AM, Guerrant RL Early childhood diarrhoea and helminthiases associate with long-term linear growth faltering. Int J Epidemiol. 2001;30(6):1457–64. 11821364 10.1093/ije/30.6.1457

[5] Dewey KG, Begum K Long-term consequences of stunting in early life. Matern Child Nutr. 2011;7 Suppl 3:5–18. 21929633 10.1111/j.1740-8709.2011.00349.x PMC6860846

[6] Schott WB, Crookston BT, Lundeen EA, Stein AD, Behrman JR Young Lives Determinants and Consequences of Child Growth Project Team. Periods of child growth up to age 8 years in Ethiopia, India, Peru and Vietnam: Key distal household and community factors. Soc Sci Med. 2013;97:278–87. 23769211 10.1016/j.socscimed.2013.05.016 PMC3812418

[7] Lunn PG, Northrop-Clewes CA, Downes RM Intestinal permeability, mucosal injury, and growth faltering in Gambian infants. Lancet Lond Engl. 1991;338(8772):907–10. 10.1016/0140-6736(91)91772-m 1681266

[8] Kosek MN, MAL-ED Network Investigators. Causal pathways from enteropathogens to environmental enteropathy: Findings from the MAL-ED Birth Cohort Study. EBioMedicine. 2017;18:109–17. 28396264 10.1016/j.ebiom.2017.02.024 PMC5405169

[9] Prendergast AJ, Kelly P Interactions between intestinal pathogens, enteropathy and malnutrition in developing countries. Curr Opin Infect Dis. 2016;29(3):229–36. 26967147 10.1097/QCO.0000000000000261 PMC4888918

[10] Watanabe K, Petri WA Environmental enteropathy: Elusive but significant subclinical abnormalities in developing countries. EBioMedicine. 2016;10:25–32. 27495791 10.1016/j.ebiom.2016.07.030 PMC5006727

[11] Locks LM, Mwiru RS, Mtisi E, Manji KP, McDonald CM, Liu E, Kupka R, Kisenge R, Aboud S, Gosselin K et al. Infant nutritional status and markers of environmental enteric dysfunction are associated with midchildhood anthropometry and blood pressure in Tanzania. J Pediatr. 2017;187:225–233.e1. 28499715 10.1016/j.jpeds.2017.04.005 PMC5533170

[12] MAL-ED Network Investigators. The MAL-ED study: A multinational and multidisciplinary approach to understand the relationship between enteric pathogens, malnutrition, gut physiology, physical growth, cognitive development, and immune responses in infants and children up to 2 years of age in resource-poor environments. Clin Infect Dis Off Publ Infect Dis Soc Am. 2014;59 Suppl 4:S193–206. 10.1093/cid/ciu653 25305287

[13] MAL-ED Network Investigators. Relationship between growth and illness, enteropathogens and dietary intakes in the first 2 years of life: Findings from the MAL-ED birth cohort study. BMJ Glob Health. 2017;2(4):e000370. 10.1136/bmjgh-2017-000370 PMC5759708 29333282

[14] MAL-ED Network Investigators. Childhood stunting in relation to the pre- and postnatal environment during the first 2 years of life: The MAL-ED longitudinal birth cohort study. PLoS Med. 2017;14(10):e1002408. 29069076 10.1371/journal.pmed.1002408 PMC5656304

[15] Richard SA, Barrett LJ, Guerrant RL, Checkley W, Miller MA, MAL-ED Network Investigators. Disease surveillance methods used in the 8-site MAL-ED cohort study. Clin Infect Dis Off Publ Infect Dis Soc Am. 2014;59 Suppl 4:S220–224. 10.1093/cid/ciu435 PMC4204606 25305290

[16] Houpt E, Gratz J, Kosek M, Zaidi AKM, Qureshi S, Kang G, Babji S, Mason C, Bodhidatta L, Samie A et al. Microbiologic methods utilized in the MAL-ED cohort study. Clin Infect Dis Off Publ Infect Dis Soc Am. 2014;59 Suppl 4:S225–232. 10.1093/cid/ciu413 PMC4204609 25305291

[17] Kosek M, Guerrant RL, Kang G, Bhutta Z, Yori PP, Gratz J, Gottlieb M, Lang D, Lee GO, Haque R et al. Assessment of environmental enteropathy in the MAL-ED cohort study: Theoretical and analytic framework. Clin Infect Dis Off Publ Infect Dis Soc Am. 2014;59 Suppl 4:S239–247. 10.1093/cid/ciu457 PMC4204611 25305293

[18] Caulfield LE, Bose A, Chandyo RK, Nesamvuni C, de Moraes ML, Turab A, Patil C, Mahfuz M, Ambikapathi R, Ahmed T et al. Infant feeding practices, dietary adequacy, and micronutrient status measures in the MAL-ED study. Clin Infect Dis Off Publ Infect Dis Soc Am. 2014;59 Suppl 4:S248–254. 10.1093/cid/ciu421 PMC4204612 25305294

[19] WHO. WHO Child Growth Standards: Methods and development: Length/height-for-age, weight-for-age, weight-for-length, weight-for-height and body mass index-for-age. Geneva: WHO; 2006.

[20] Richard SA, McCormick BJJ, Seidman JC, Rasmussen Z, Kosek MN, Rogawski ET, Petri W, Bose A, Mduma E, Maciel BLL et al. Relationships among common illness symptoms and the protective effect of breastfeeding in early childhood in MAL-ED: An eight-country cohort study. Am J Trop Med Hyg. 2018;98(3):904–12. 29380724 10.4269/ajtmh.17-0457 PMC5930868

[21] Platts-Mills JA, Babji S, Bodhidatta L, Gratz J, Haque R, Havt A, McCormick BJJ, McGrath M, Olortegui MP, Samie A et al. Pathogen-specific burdens of community diarrhoea in developing countries: A multisite birth cohort study (MAL-ED). Lancet Glob Health. 2015;3(9):e564–575. 26202075 10.1016/S2214-109X(15)00151-5 PMC7328884

[22] Liu J, Platts-Mills JA, Juma J, Kabir F, Nkeze J, Okoi C, Operario DJ, Uddin J, Ahmed S, Alonso PL et al. Use of quantitative molecular diagnostic methods to identify causes of diarrhoea in children: A reanalysis of the GEMS case–control study. Lancet Lond Engl. 2016;388(10051):1291–301. 10.1016/S0140-6736(16)31529-X PMC5471845 27673470

[23] Kosek MN, Lee GO, Guerrant RL, Haque R, Kang G, Ahmed T, Bessong P, Ali A, Mduma E, Yori PP et al. Age and sex normalization of intestinal permeability measures for the improved assessment of enteropathy in infancy and early childhood: Results from the MAL-ED Study. J Pediatr Gastroenterol Nutr. 2017;65(1):31–9. 28644347 10.1097/MPG.0000000000001610

[24] McCormick BJJ, Lee GO, Seidman JC, Haque R, Mondal D, Quetz J, Lima AAM, Babji S, Kang G, Shrestha SK et al. Dynamics and trends in fecal biomarkers of gut function in children from 1–24 Months in the MAL-ED Study. Am J Trop Med Hyg. 2017;96(2):465–72. 27994110 10.4269/ajtmh.16-0496 PMC5303054

[25] Willett W, Stampfer MJ Total energy intake: Implications for epidemiologic analyses. Am J Epidemiol. 1986;124(1):17–27. 3521261 10.1093/oxfordjournals.aje.a114366

[26] Namaste SM, Rohner F, Huang J, Bhushan NL, Flores-Ayala R, Kupka R, Mei Z, Rawat R, Williams AM, Raiten DJ et al. Adjusting ferritin concentrations for inflammation: Biomarkers Reflecting Inflammation and Nutritional Determinants of Anemia (BRINDA) project. Am J Clin Nutr. 2017;106(Suppl 1):359S–71S. 28615259 10.3945/ajcn.116.141762 PMC5490647

[27] Rohner F, Namaste SM, Larson LM, Addo OY, Mei Z, Suchdev PS, Williams AM, Ashour FAS, Rawat R, Raiten DJ et al. Adjusting soluble transferrin receptor concentrations for inflammation: Biomarkers Reflecting Inflammation and Nutritional Determinants of Anemia (BRINDA) project. Am J Clin Nutr. 2017;106(Suppl 1):372S–82S. 28615256 10.3945/ajcn.116.142232 PMC5490651

[28] Psaki SR, Seidman JC, Miller M, Gottlieb M, Bhutta ZA, Ahmed T, Ahmed AS, Bessong P, John SM, Kang G et al. Measuring socio-economic status in multicountry studies: Results from the eight-country MAL-ED study. Popul Health Metr. 2014;12(1):8. 24656134 10.1186/1478-7954-12-8 PMC4234146

[29] Harper KM, Mutasa M, Prendergast AJ, Humphrey J, Manges AR Environmental enteric dysfunction pathways and child stunting: A systematic review. PLoS Negl Trop Dis. 2018;12(1):e0006205. 29351288 10.1371/journal.pntd.0006205 PMC5792022

[30] GBD 2016 Diarrhoeal Disease Collaborators. Estimates of the global, regional, and national morbidity, mortality, and aetiologies of diarrhoea in 195 countries: A systematic analysis for the Global Burden of Disease Study 2016. Lancet Infect Dis. 2018;18(11):1211–28. 30243583 10.1016/S1473-3099(18)30362-1 PMC6202444

[31] Lim SS, Vos T, Flaxman AD, Danaei G, Shibuya K, Adair-Rohani H, Amann M, Anderson HR, Andrews KG, Aryee M et al. A comparative risk assessment of burden of disease and injury attributable to 67 risk factors and risk factor clusters in 21 regions, 1990–2010: A systematic analysis for the Global Burden of Disease Study 2010. Lancet Lond Engl. 2012;380(9859):2224–60. 10.1016/S0140-6736(12)61766-8 PMC4156511 23245609

[32] Poskitt EM, Cole TJ, Whitehead RG Less diarrhoea but no change in growth: 15 years’ data from 3 Gambian villages. Arch Dis Child. 1999;80(2):115–9.; discussion 119–120. 10325724 10.1136/adc.80.2.115 PMC1717825

[33] Stevens GA, Finucane MM, Paciorek CJ, Flaxman SR, White RA, Donner AJ, Ezzati M Trends in mild, moderate, and severe stunting and underweight, and progress towards MDG 1 in 141 developing countries: A systematic analysis of population representative data. Lancet Lond Engl. 2012;380(9844):824–34. 10.1016/S0140-6736(12)60647-3 PMC3443900 22770478

[34] Wang W, Knovich MA, Coffman LG, Torti FM, Torti SV Serum ferritin: Past, present and future. Biochim Biophys Acta. 2010;1800(8):760–9. 20304033 10.1016/j.bbagen.2010.03.011 PMC2893236

[35] Macedo MF, de Sousa M Transferrin and the transferrin receptor: Of magic bullets and other concerns. Inflamm Allergy Drug Targets. 2008;7(1):41–52. 18473900 10.2174/187152808784165162

[36] Lönnerdal B Development of iron homeostasis in infants and young children. Am J Clin Nutr. 2017;106(Suppl 6):1575S–80S. 29070561 10.3945/ajcn.117.155820 PMC5701726

[37] Nataro JP, Guerrant RL Chronic consequences on human health induced by microbial pathogens: Growth faltering among children in developing countries. Vaccine. 2017;35(49 Pt A):6807–12. 28549806 10.1016/j.vaccine.2017.05.035

[38] DeBoer MD, Chen D, Burt DR, Ramirez-Zea M, Guerrant RL, Stein AD, Martorell R, Luna MA Early childhood diarrhea and cardiometabolic risk factors in adulthood: The Institute of Nutrition of Central America and Panama Nutritional Supplementation Longitudinal Study. Ann Epidemiol. 2013;23(6):314–20. 23608305 10.1016/j.annepidem.2013.03.012 PMC4431615

[39] Lee GO, Olortegui MP, Salas MS, Yori PP, Trigoso DR, Kosek P, Mispireta ML, Oberhelman R, Caulfield LE, Kosek MN Environmental enteropathy is associated with cardiometabolic risk factors in Peruvian children. J Dev Orig Health Dis. 2017;8(3):337–48. 28264759 10.1017/S2040174417000071

