# Supplementary material for: Enteric dysfunction and other factors associated with attained size at 5 years: MAL-ED birth cohort study findings
Source: Am J Clin Nutr. 2019 May 25;110(1):131–8. doi: 10.1093/ajcn/nqz004 (PMC6599740; doi:10.1093/ajcn/nqz004)

Supplemental Table 1. Comparison of children retained in the cohort through five years of age (‘In’) and those lost-to-follow up or with incomplete data (‘Out’).

|  | Maternal age^a^ | | Maternal education^a^ | | Mean WAMI^a^ | | LAZ at enrolment^a^ | | WAZ at enrolment^a^ | | WLZ at enrolment^a^ | | Sex (% boys)^b^ | |
| --- | --- | --- | --- | --- | --- | --- | --- | --- | --- | --- | --- | --- | --- | --- |
| Site | Out | In | Out | In | Out | In | Out | In | Out | In | Out | In | Out | In |
| BGD | 24.5 | 25.0 | 4.3 | 4.7 | 0.5 | 0.5 | -1.3 | -1.0* | -1.4 | -1.3 | -0.7 | -1.0* | 43 | 51 |
| NEB | 23.8 | 23.9 | 7.9 | 6.8 | 0.5 | 0.5 | -1.2 | -1.0 | -1.5 | -1.3 | -1.1 | -1.2 | 43 | 45 |
| INV | 26.4 | 26.5 | 8.0 | 8.5 | 0.7 | 0.7 | -0.8 | -0.7 | -1.0 | -0.9 | -0.9 | -0.9 | 63 | 46* |
| BRF | 23.9 | 26.0* | 9.2 | 9.1 | 0.8 | 0.8 | -0.9 | -0.8 | -0.2 | -0.1 | 0.4 | 0.5 | 46 | 59 |
| PEL | 23.8 | 24.6 | 7.9 | 7.6 | 0.6 | 0.5 | -0.9 | -1.0 | -0.6 | -0.7 | -0.1 | -0.0 | 51 | 54 |
| SAV | 25.9 | 27.1 | 10.4 | 10.1 | 0.8 | 0.8 | -0.8 | -0.8 | -0.5 | -0.4 | -0.1 | 0.0 | 47 | 52 |
| TZH | 27.6 | 29.3* | 5.1 | 5.1 | 0.2 | 0.2 | -1.1 | -1.0 | -0.2 | -0.2 | 0.8 | 0.7 | 50 | 48 |
| Overall | 25.3 | 25.8 | 7.9 | 7.1* | 0.59 | 0.57* | -0.9 | -0.9 | -0.6 | -0.8* | -0.1 | -0.4* | 50 | 50 |

^a^ T-test performed

^b^ Chi-squared test performed

* P<0.05

Supplemental Table 2. Candidate Variables

| Baseline variables |  |  |
| --- | --- | --- |
| Variable | Description | Type |
| education | Baseline maternal education | Continuous |
| edb | Primary school education at baseline | Binary |
| waze | WAZ at enrollment | Continuous |
| laze | LAZ at enrollment | Continuous |
| mafht | Maternal height | Continuous |
| bmi | Maternal BMI | Continuous |
| ma0fe1 | Sex | Binary |
| par1 | First born to mother | Binary |
|  |  |  |
| Summarized SES/environmental variables (over 2y, ≥1 assessment) | |  |
| meanwami | Mean WAMI * 10 | Continuous |
| mnppr | Mean crowding | Continuous |
| meants | Mean food security score | Continuous |
| impsan | Improved sanitation >50% of time | Binary |
|  |  |  |
| HOME, SRQ, Ravens variables | |  |
| f1_evrhit6 | HOME emotional factor 6m | Continuous |
| f1_evrhit24 | HOME emotional factor 24m | Continuous |
| f2_eshit6 | HOME safety factor 6m | Continuous |
| f2_eshit24 | HOME safety factor 24m | Continuous |
| f3_chit6 | HOME cleanliness factor 6m | Continuous |
| f3_chit24 | HOME cleanliness factor 24m | Continuous |
| tot_score16srq1 | SRQ 1m | Continuous |
| tot_score16srq15 | SRQ 15m | Continuous |
| tot_score16srq24 | SRQ 24m | Continuous |
| f1_raventotrcm | Raven's factor | Continuous |
|  |  |  |
| Summarized illness variables (prevalence and incidence, first 2y, require ≥700 days) | |  |
| dprev3p | Days with diarrhea per year in the first 2 years of life | Continuous |
| dint3p | Episodes of diarrhea per year in the first 2 years of life | Continuous |
| aint3p | Episodes of ALRI per year in the first 2 years of life | Continuous |
| sevdp | Days with severe diarrhea per year in the first 2 years of life | Continuous |
| persp | Days with persistent diarrhea per year in the first 2 years of life | Continuous |
| safabp | Days with antibiotics per year in the first 2 years of life | Continuous |
| safillp | Days with maternally reported illness per year in the first 2 years of life | Continuous |
| saffevp | Days with fever per year in the first 2 years of life | Continuous |
| *with 6 at end for the early exposure variables (<6m) | |  |
| ** with y2 at end for the 25-36m time period | |  |
|  |  |  |
| Summarized diet variables (9-24m, ≥11 assessments, 25-36m ≥4 assessments) | |  |
| sumkcal_bcT | Standardized (t-score) mean total energy (9-24m) | Continuous |
| procalT | Mean protein density (9-24m) | Continuous |
| aprocalT | Mean animal source protein density (9-24m) | Continuous |
| mfpprocalT | Mean meat/fish/poultry protein density (9-24m) | Continuous |
| folcalT | Mean folate density (9-24m) | Continuous |
| fatcalT | Mean fat density (9-24m) | Continuous |
| fapucalT | Mean poly-unsaturated fat density (9-24m) | Continuous |
| fasatcalT | Mean saturated fat density (9-24m) | Continuous |
| famscalT | Mean mono-unsaturated fat density (9-24m) | Continuous |
| cholecalT | Mean cholesterol density (9-24m) | Continuous |
| chocalT | Mean carbohydrate density (9-24m) | Continuous |
| fecalT | Mean iron density (9-24m) | Continuous |
| nacalT | Mean sodium density (9-24m) | Continuous |
| zncalT | Mean zinc density (9-24m) | Continuous |
| mncalT | Mean manganese density (9-24m) | Continuous |
| cacalT | Mean calcium density (9-24m) | Continuous |
| k_calT | Mean potassium density (9-24m) | Continuous |
| mgcalT | Mean magnesium density (9-24m) | Continuous |
| vitacalT | Mean vitamin A density (9-24m) | Continuous |
| vitccalT | Mean vitamin C density (9-24m) | Continuous |
| vitecalT | Mean vitamin E density (9-24m) | Continuous |
| vitb6calT | Mean vitamin B6 density (9-24m) | Continuous |
| vitb12calT | Mean vitamin B12 density (9-24m) | Continuous |
| vitdcalT | Mean vitamin D density (9-24m) | Continuous |
| phytatecalT | Mean phytate density (9-24m) | Continuous |
| *Variables for the 25-36m period end in '2' | |  |
|  |  |  |
| Breastfeeding (≥700 days of data) | |  |
| minasolid | Earliest age of solid foods | Continuous |
| minanobf2 | Age after which no more breast milk is reported (or last day of breastfeeding data) | Continuous |
|  |  |  |
| Blood (≥1 assessment) | |  |
| hb_adj | Hemoglobin (mean of 1-3 measurements) | Continuous |
| adjzinc_mml | Zinc (mean of 1-3 measurements) | Continuous |
| adjrar | Retinol (mean of 1-3 measurements) (square root) | Continuous |
| adjfar | Ferritin (mean of 1-3 measurements) (square root) | Continuous |
| adjtfr | TfR (mean of 1-3 measurements) (square root) | Continuous |
| bllconc | Lead (1 measurement) | Continuous |
| agpval1 | AGP (mean of 1-3 measurements) | Continuous |
| highagp | Number of times with high AGP | Continuous |
| anemia | Number of times with anemia | Continuous |
| lowadjzinc | Number of times with low zinc | Continuous |
| lowadjrar | Number of times with low retinol | Continuous |
| highagpb | Ever high AGP | Binary |
| anemiab | Ever anemia | Binary |
| lowadjzincb | Ever low zinc | Binary |
| lowadjrarb | Ever low retinol | Binary |
| highbll | High blood lead level | Binary |
|  |  |  |
| EE (≥8 assessments) |  |  |
| alab | Mean log concentration Alpha-1 antitrypsin (detrended) | Continuous |
| mpob | Mean log concentration Myeloperoxidase (detrended) | Continuous |
| neob | Mean log concentration Neopterin (detrended) | Continuous |
|  |  |  |
| LMZ (≥3 assessments) | |  |
| lmz | Mean LMZ | Continuous |
|  |  |  |
| Pathogen variables* | Proportion monthly stool samples with any… (≥9 samples for the 2y data) |  |
| sumpathg.nonrvdens | pathogens (total # pathogens / total # stools) | Continuous |
| path1dens | virus (total # viruses / total # stools * 10) (beta ~ increase of 10% in virus density) | Continuous |
| path2dens | bacteria (total # bacteria / total # stools * 10) (beta ~ increase of 10% in bacteria density) | Continuous |
| path3dens | ETEC (st and lt) (beta ~ increase of 10% in pathogen density) | Continuous |
| atypicalepecdens | atypical epec (beta ~ increase of 10% in pathogen density) | Continuous |
| lt.etecdens | lt_etec (beta ~ increase of 10% in pathogen density) | Continuous |
| epecdens | epec (beta ~ increase of 10% in pathogen density) | Continuous |
| adenodens | adenovirus (beta ~ increase of 10% in pathogen density) | Continuous |
| astrodens | astrovirus (beta ~ increase of 10% in pathogen density) | Continuous |
| rotadens | rotavirus (beta ~ increase of 10% in pathogen density) | Continuous |
| campydens | campylobacter (beta ~ increase of 10% in pathogen density) | Continuous |
| st.etecdens | st_etec (beta ~ increase of 10% in pathogen density) | Continuous |
| eaecdens | EAEC (beta ~ increase of 10% in pathogen density) | Continuous |
| pshigyndens | plesiomonas shigilloides (beta ~ increase of 10% in pathogen density) | Continuous |
| giarddens | Giardia (beta ~ increase of 10% in pathogen density) | Continuous |
| cryptodens | cryptosporidium (beta ~ increase of 10% in pathogen density) | Continuous |
| *also considered diarrheal stools, and early (<6 months) exposures | |  |

Supplemental Table 3. Univariate models of factors in early childhood associated with growth to five years of age (including height-for-age (HAZ), weight-for-age (WAZ), body mass index (BMIZ), and weight-for-height (WHZ) z-score) (standard deviation). The models include site as a dummy variable (results not shown).

|  | HAZ | WAZ | BMIZ | WHZ |  |  |
| --- | --- | --- | --- | --- | --- | --- |
| WAMI | 0.16 (0.02)^***^ | 0.17 (0.03)^***^ | 0.09 (0.03) ^***^ | 0.10 (0.03)^***^ |  |  |
| Sex (boys 0, girls 1) | 0.01 (0.06) | -0.08 (0.06) | -0.17 (0.07)* | -0.07 (0.07) |  |  |
| Maternal height (cm) | 0.05 (0.00)^***^ | 0.04 (0.01)^***^ | 0.01 (0.01) | 0.01 (0.01) |  |  |
| Energy intake^1^ | 0.10 (0.03)^***^ | 0.12 (0.03)^***^ | 0.08 (0.03)^*^ | 0.08 (0.04)^*^ |  |  |
| Protein density^1^ | 0.07 (0.03)^**^ | 0.05 (0.03) | -0.00 (0.03) | -0.00 (0.04) |  |  |
| Haemoglobin | 0.08 (0.03)^**^ | 0.06 (0.03) | 0.01 (0.03) | 0.01 (0.03) |  |  |
| Transferrin receptor^2^ | 0.10 (0.06) | 0.13 (0.07) | 0.10 (0.07) | 0.10 (0.08) |  |  |
| Ferritin^2^ | -0.05 (0.02)^**^ | -0.03 (0.02) | 0.01 (0.02) | 0.01 (0.02) |  |  |
| AGP | -0.01 (0.01) | 0.00 (0.01) | 0.01 (0.01) | 0.01 (0.01) |  |  |
| Bacterial density | -0.07 (0.02)^***^ | -0.05 (0.02)^**^ | -0.01 (0.02) | -0.01 (0.02) |  |  |
| Lactulose-Mannitol Z-score^a^ | -0.17 (0.05)^***^ | -0.20 (0.05)^***^ | -0.12 (0.06)^*^ | -0.15 (0.06)^**^ |  |  |
| Alpha-1-antitrypsin ^3^ | -0.48 (0.13)^***^ | -0.50 (0.15)^***^ | -0.24 (0.16) | -0.26 (0.16) |  |  |
| Myeloperoxidase ^3^ | -0.40 (0.12)^***^ | -0.66 (0.13)^***^ | -0.60 (0.14)^***^ | -0.63 (0.15)^***^ |  |  |
| LAZ at enrolment | 0.30 (0.03)^***^ |  | 0.06 (0.03) |  |  |  |
| WAZ at enrolment |  | 0.27 (0.03) ^***^ |  |  |  |  |
| WLZ at enrolment |  |  |  | 0.17 (0.03) ^***^ |  |  |
| ^***^p < 0.001, ^**^p < 0.01, ^*^p < 0.05  ^1^: Variables have been standardized – The beta estimates represent the difference in one standard deviation change in the variable;  ^2^: Variables have been adjusted for inflammation and normalized by taking the square root;  ^3^: Mean log concentration (detrended) | | | | | | |

Supplemental Table 4. Factors in early childhood associated with linear growth (height-for-age z-score) to five years of age. The ‘base model’ does not include indicators of gut function, whereas the ‘gut function’ model does not include the diet, micronutrient status or pathogen variables. The ‘final’ model is then shown to indicate the comparison between coefficients when variables from the two models are combined. (standard deviation) The models include site as a dummy variable (results not shown).

|  | **Base model** | **Gut function** | **Final** |
| --- | --- | --- | --- |
| LAZ at enrolment | 0.27 (0.02)^***^ | 0.26 (0.02)^***^ | 0.28 (0.02)^***^ |
| WAMI | 0.11 (0.02)^***^ | 0.11 (0.02)^***^ | 0.10 (0.02)^***^ |
| Sex (boys 0, girls 1) | 0.05 (0.05) | -0.01 (0.05) | 0.04 (0.05) |
| Maternal height (cm) | 0.03 (0.00)^***^ | 0.03 (0.00)^***^ | 0.03 (0.00)^***^ |
| Energy intake | 0.05 (0.03)^*^ |  | 0.03 (0.03) |
| Protein density | 0.03 (0.03) |  | 0.03 (0.03) |
| Haemoglobin | 0.08 (0.03)^**^ |  | 0.06 (0.03)^*^ |
| Transferrin receptor | 0.18 (0.06)^**^ |  | 0.18 (0.06)^**^ |
| Ferritin | -0.08 (0.02)^***^ |  | -0.08 (0.02)^***^ |
| Bacterial density | -0.04 (0.02)^*^ |  | -0.04 (0.02)^*^ |
| Lactulose-Mannitol Z-score |  | -0.13 (0.04)^**^ | -0.11 (0.04)^**^ |
| Alpha-1-antitrypsin |  | -0.30 (0.13)^*^ | -0.28 (0.12)^*^ |
| Myeloperoxidase |  | -0.23 (0.11)^*^ | -0.19 (0.11) |
| R^2^ | 0.41 | 0.39 | 0.42 |
| Adj. R^2^ | 0.40 | 0.38 | 0.41 |
| Num. obs. | 1017 | 1017 | 1017 |
| RMSE | 0.79 | 0.80 | 0.78 |
| ^***^p < 0.001, ^**^p < 0.01, ^*^p < 0.05 | |  |  |

Supplemental Table 5. Comparison of LMZ scores at each time point and linear growth (height-for-age z-score) at five years of age. (standard deviation) The models include site as a dummy variable (results not shown).

|  | **Final** | **LMZ 3m** | **LMZ 6m** | **LMZ 9m** | | **LMZ 15m** | |
| --- | --- | --- | --- | --- | --- | --- | --- |
| LAZ at enrolment | 0.28 (0.02)*** | 0.26 (0.03)*** | 0.28 (0.03)*** | 0.27 (0.03)*** | | 0.28 (0.03)*** | |
| WAMI | 0.10 (0.02)*** | 0.10 (0.02)*** | 0.09 (0.02)*** | 0.10 (0.02)*** | | 0.09 (0.02)*** | |
| Sex (boys 0, girls 1) | 0.04 (0.05) | 0.00 (0.05) | 0.05 (0.06) | 0.04 (0.05) | | 0.08 (0.05) | |
| Maternal height (cm) | 0.03 (0.00)*** | 0.04 (0.00)*** | 0.03 (0.01)*** | 0.03 (0.01)*** | | 0.03 (0.00)*** | |
| Energy intake | 0.03 (0.03) | 0.02 (0.03) | 0.03 (0.03) | 0.03 (0.03) | | 0.04 (0.03) | |
| Protein density | 0.03 (0.03) | 0.04 (0.03) | 0.02 (0.03) | 0.03 (0.03) | | 0.04 (0.03) | |
| Haemoglobin | 0.06 (0.03)* | 0.06 (0.03)* | 0.06 (0.03)* | 0.07 (0.03)* | | 0.06 (0.03)* | |
| Transferrin receptor | 0.18 (0.06)** | 0.24 (0.07)*** | 0.17 (0.07)* | 0.19 (0.07)** | | 0.18 (0.07)** | |
| Ferritin | -0.08 (0.02)*** | -0.06 (0.02)** | -0.09 (0.02)*** | -0.08 (0.02)*** | | -0.08 (0.02)*** | |
| Bacterial density | -0.04 (0.02)* | -0.05 (0.02)** | -0.04 (0.02)* | -0.03 (0.02) | | -0.04 (0.02)* | |
| Alpha-1-antitrypsin | -0.28 (0.12)* | -0.23 (0.13) | -0.23 (0.14) | -0.21 (0.13) | | -0.32 (0.14)* | |
| Myeloperoxidase | -0.19 (0.11) | -0.22 (0.12) | -0.24 (0.12)* | -0.25 (0.12)* | | -0.20 (0.12) | |
| LMZ (mean 3-15m) | -0.11 (0.04)** |  |  |  | |  | |
| LMZ 3m |  | -0.04 (0.03) |  |  | |  | |
| LMZ 6m |  |  | -0.06 (0.03) |  | |  | |
| LMZ 9m |  |  |  | -0.07 (0.03)* | |  | |
| LMZ 15m |  |  |  |  | | -0.01 (0.03) | |
| R^2^ | 0.42 | 0.41 | 0.40 | 0.40 | | 0.40 | |
| Adj. R^2^ | 0.41 | 0.40 | 0.38 | 0.39 | | 0.39 | |
| Num. obs. | 1017 | 882 | 885 | 895 | | 904 | |
| RMSE | 0.78 | 0.77 | 0.79 | 0.79 | | 0.79 | |
| ***p < 0.001, **p < 0.01, *p < 0.05 | | | |  |  | |  |

Supplemental Table 6. Comparison of lactulose scores at each time point and linear growth (height-for-age z-score) at five years of age. (standard deviation) The models include site as a dummy variable (results not shown).

|  | **Final** | **Lac 3m** | **Lac 6m** | | **Lac 9m** | | | **Lac 15m** | |
| --- | --- | --- | --- | --- | --- | --- | --- | --- | --- |
| LAZ at enrolment | 0.28 (0.02)^***^ | 0.26 (0.03)^***^ | 0.28 (0.03)^***^ | | 0.27 (0.03)^***^ | | | 0.28 (0.03)^***^ | |
| WAMI | 0.10 (0.02)^***^ | 0.11 (0.02)^***^ | 0.09 (0.02)^***^ | | 0.10 (0.02)^***^ | | | 0.09 (0.02)^***^ | |
| Sex (boys 0, girls 1) | 0.07 (0.05) | 0.01 (0.05) | 0.09 (0.06) | | 0.06 (0.05) | | | 0.09 (0.05) | |
| Maternal height (cm) | 0.03 (0.00)^***^ | 0.04 (0.00)^***^ | 0.03 (0.01)^***^ | | 0.03 (0.01)^***^ | | | 0.03 (0.00)^***^ | |
| Energy intake | 0.04 (0.03) | 0.02 (0.03) | 0.04 (0.03) | | 0.03 (0.03) | | | 0.04 (0.03) | |
| Protein density | 0.03 (0.03) | 0.04 (0.03) | 0.02 (0.03) | | 0.03 (0.03) | | | 0.03 (0.03) | |
| Haemoglobin | 0.07 (0.03)^*^ | 0.06 (0.03)^*^ | 0.06 (0.03)^*^ | | 0.07 (0.03)^**^ | | | 0.06 (0.03)^*^ | |
| Transferrin receptor | 0.18 (0.06)^**^ | 0.25 (0.07)^***^ | 0.17 (0.07)^**^ | | 0.19 (0.07)^**^ | | | 0.18 (0.07)^**^ | |
| Ferritin | -0.08 (0.02)^***^ | -0.06 (0.02)^**^ | -0.09 (0.02)^***^ | | -0.09 (0.02)^***^ | | | -0.09 (0.02)^***^ | |
| Bacterial density | -0.04 (0.02)^*^ | -0.05 (0.02)^**^ | -0.04 (0.02)^*^ | | -0.03 (0.02) | | | -0.04 (0.02)^*^ | |
| Alpha-1-antitrypsin | -0.29 (0.12)^*^ | -0.22 (0.13) | -0.23 (0.14) | | -0.23 (0.14) | | | -0.31 (0.14)^*^ | |
| Myeloperoxidase | -0.20 (0.11) | -0.25 (0.12)^*^ | -0.25 (0.12) ^*^ | | -0.25 (0.12)^*^ | | | -0.21 (0.12) | |
| Lactulose | -0.06 (0.03) |  |  | |  | | |  | |
| Lactulose 3m |  | 0.00 (0.02) |  | |  | | |  | |
| Lactulose 6m |  |  | -0.05 (0.02)^*^ | |  | | |  | |
| Lactulose 9m |  |  |  | | -0.03 (0.03) | | |  | |
| Lactulose 15m |  |  |  | |  | | | -0.02 (0.02) | |
| R^2^ | 0.41 | 0.41 | 0.40 | | 0.40 | | | 0.40 | |
| Adj. R^2^ | 0.40 | 0.40 | 0.39 | | 0.39 | | | 0.39 | |
| Num. obs. | 1017 | 878 | 880 | | 895 | | | 905 | |
| RMSE | 0.78 | 0.77 | 0.79 | | 0.79 | | | 0.79 | |
| ***p < 0.001, **p < 0.01, *p < 0.05 | | | |  | |  |  | |  |

Supplemental Table 7. Comparison of mannitol scores at each time point and linear growth (height-for-age z-score) at five years of age. (standard deviation) The models include site as a dummy variable (results not shown).

|  | **Final** | **M 3m** | **M 6m** | | **M 9m** | | | **M 15m** | |
| --- | --- | --- | --- | --- | --- | --- | --- | --- | --- |
| LAZ at enrolment | 0.28 (0.02)^***^ | 0.26 (0.03)^***^ | 0.28 (0.03)^***^ | | 0.27 (0.03)^***^ | | | 0.28 (0.03)^***^ | |
| WAMI | 0.10 (0.02)^***^ | 0.10 (0.02)^***^ | 0.10 (0.02)^***^ | | 0.10 (0.02)^***^ | | | 0.09 (0.02)^***^ | |
| Sex (boys 0, girls 1) | 0.05 (0.05) | 0.00 (0.05) | 0.07 (0.06) | | 0.05 (0.05) | | | 0.08 (0.05) | |
| Maternal height (cm) | 0.03 (0.00)^***^ | 0.04 (0.00)^***^ | 0.03 (0.01)^***^ | | 0.03 (0.01)^***^ | | | 0.03 (0.00)^***^ | |
| Energy intake | 0.04 (0.03) | 0.02 (0.03) | 0.03 (0.03) | | 0.03 (0.03) | | | 0.04 (0.03) | |
| Protein density | 0.03 (0.03) | 0.04 (0.03) | 0.02 (0.03) | | 0.03 (0.03) | | | 0.03 (0.03) | |
| Haemoglobin | 0.07 (0.03)^**^ | 0.06 (0.03)^*^ | 0.06 (0.03)^*^ | | 0.07 (0.03)^**^ | | | 0.06 (0.03)^*^ | |
| Transferrin receptor | 0.18 (0.06)^**^ | 0.24 (0.07)^***^ | 0.17 (0.07)^*^ | | 0.18 (0.07)^**^ | | | 0.18 (0.07)^**^ | |
| Ferritin | -0.08 (0.02)^***^ | -0.06 (0.02)^**^ | -0.09 (0.02)^***^ | | -0.09 (0.02)^***^ | | | -0.08 (0.02)^***^ | |
| Bacterial density | -0.04 (0.02)^*^ | -0.05 (0.02)^**^ | -0.04 (0.02)^*^ | | -0.03 (0.02) | | | -0.04 (0.02)^*^ | |
| Alpha-1-antitrypsin | -0.29 (0.12)^*^ | -0.23 (0.13) | -0.23 (0.14) | | -0.24 (0.13) | | | -0.32 (0.14)^*^ | |
| Myeloperoxidase | -0.20 (0.11) | -0.22 (0.12) | -0.25 (0.12)^*^ | | -0.26 (0.12)^*^ | | | -0.20 (0.12) | |
| Mannitol | 0.02 (0.04) |  |  | |  | | |  | |
| Mannitol 3m |  | 0.02 (0.03) |  | |  | | |  | |
| Mannitol 6m |  |  | -0.03 (0.03) | |  | | |  | |
| Mannitol 9m |  |  |  | | 0.03 (0.03) | | |  | |
| Mannitol 15m |  |  |  | |  | | | -0.00 (0.02) | |
| R^2^ | 0.41 | 0.41 | 0.40 | | 0.40 | | | 0.40 | |
| Adj. R^2^ | 0.40 | 0.40 | 0.38 | | 0.39 | | | 0.39 | |
| Num. obs. | 1017 | 883 | 886 | | 896 | | | 906 | |
| RMSE | 0.78 | 0.77 | 0.79 | | 0.79 | | | 0.79 | |
| ***p < 0.001, **p < 0.01, *p < 0.05 | | | |  | |  |  | |  |

Supplemental Table 8. Factors in early childhood associated with somatic growth (weight-for-age z-score) to five years of age. The ‘base model’ does not include indicators of gut function, whereas the ‘gut function’ model does not include the diet, micronutrient status or pathogen variables. The ‘final’ model is then shown to indicate the comparison between coefficients when variables from the two models are combined. (standard deviation) The models include site as a dummy variable (results not shown).

|  | **Base model** | | | | | **Gut function** | | **Final** | | |  |
| --- | --- | --- | --- | --- | --- | --- | --- | --- | --- | --- | --- |
| WAZ at enrolment | | 0.25 (0.03)^***^ | | 0.25 (0.03)^***^ | | | 0.27 (0.03)^***^ | | | | |
| WAMI | | 0.13 (0.03)^***^ | | 0.11 (0.02)^***^ | | | 0.11 (0.03)^***^ | | | | |
| Sex (boys 0, girls 1) | | -0.04 (0.06) | | -0.10 (0.06) | | | -0.05 (0.06) | | | | |
| Maternal height (cm) | | 0.03 (0.01)^***^ | | 0.03 (0.01)^***^ | | | 0.03 (0.01)^***^ | | | | |
| Energy intake | | 0.08 (0.03)^*^ | |  | | | 0.05 (0.03) | | | | |
| Protein density | | 0.01 (0.03) | |  | | | 0.00 (0.03) | | | | |
| Haemoglobin | | 0.04 (0.03) | |  | | | 0.02 (0.03) | | | | |
| Transferrin receptor | | 0.21 (0.07)^**^ | |  | | | 0.21 (0.07)^**^ | | | | |
| Ferritin | | -0.05 (0.02)^*^ | |  | | | -0.05 (0.02)^*^ | | | | |
| Bacterial density | | -0.02 (0.02) | |  | | | -0.02 (0.02) | | | | |
| Lactulose-Mannitol Z-score | |  | | -0.16 (0.05)^***^ | | | -0.16 (0.05)^***^ | | | | |
| Alpha-1-antitrypsin | |  | | -0.22 (0.15) | | | -0.20 (0.15) | | | | |
| Myeloperoxidase | |  | | -0.54 (0.13)^***^ | | | -0.52 (0.13)^***^ | | | | |
| R^2^ | | | 0.36 | 0.37 | | | | 0.39 | |  |  |
| Adj. R^2^ | | | 0.36 | 0.37 | | | | 0.38 | |  |  |
| Num. obs. | | | 1017 | 1017 | | | | 1017 | |  |  |
| RMSE | | | 0.94 | 0.93 | | | | 0.92 | |  |  |
| ^***^p < 0.001, ^**^p < 0.01, ^*^p < 0.05 | | |  | |  | | |  |  |  |  |

Supplemental Table 9. Factors in early childhood associated with ponderal growth (BMI z-score) to five years of age. The ‘base model’ does not include indicators of gut function, whereas the ‘gut function’ model does not include the diet, micronutrient status or pathogen variables. The ‘final’ model is then shown to indicate the comparison between coefficients when variables from the two models are combined. (standard deviation) The models include site as a dummy variable (results not shown).

|  | **Base model** | **Gut function** | **Final** |
| --- | --- | --- | --- |
| LAZ at enrolment | 0.06 (0.03) | 0.06 (0.03) | 0.06 (0.03) |
| WAMI | 0.08 (0.03)^**^ | 0.07 (0.03)^*^ | 0.07 (0.03)^*^ |
| Sex (boys 0, girls 1) | -0.16 (0.07)^*^ | -0.18 (0.07)^**^ | -0.18 (0.07)^*^ |
| Maternal height (cm) | 0.00 (0.01) | 0.00 (0.01) | 0.00 (0.01) |
| Energy intake | 0.05 (0.04) |  | 0.03 (0.04) |
| Protein density | -0.02 (0.03) |  | -0.03 (0.03) |
| Haemoglobin | 0.01 (0.03) |  | -0.01 (0.03) |
| Transferrin receptor | 0.14 (0.08) |  | 0.14 (0.08) |
| Ferritin | 0.02 (0.03) |  | 0.02 (0.03) |
| Bacterial density | 0.01 (0.02) |  | 0.01 (0.02) |
| Lactulose-Mannitol Z- score |  | -0.10 (0.06) | -0.11 (0.06)^*^ |
| Alpha-1-antitrypsin |  | 0.04 (0.17) | 0.06 (0.17) |
| Myeloperoxidase |  | -0.56 (0.15)^***^ | -0.56 (0.15)^***^ |
| R2 | 0.22 | 0.23 | 0.23 |
| Adj. R2 | 0.21 | 0.22 | 0.22 |
| Num. obs. | 1017 | 1017 | 1017 |
| RMSE | 1.06 | 1.05 | 1.05 |
| ***p < 0.001, **p < 0.01, *p < 0.05 | | |  |

Supplemental Figure 1. MAL-ED cohort profile of children who were retained in the analysis. [Sites: BGD: Bangladesh – Dhaka; INV: India – Vellore; NEB: Nepal – Bhaktapur; BRF: Brazil – Fortaleza; PEL: Peru – Loreto; SAV: South Africa – Venda; TZH: Tanzania – Haydom]


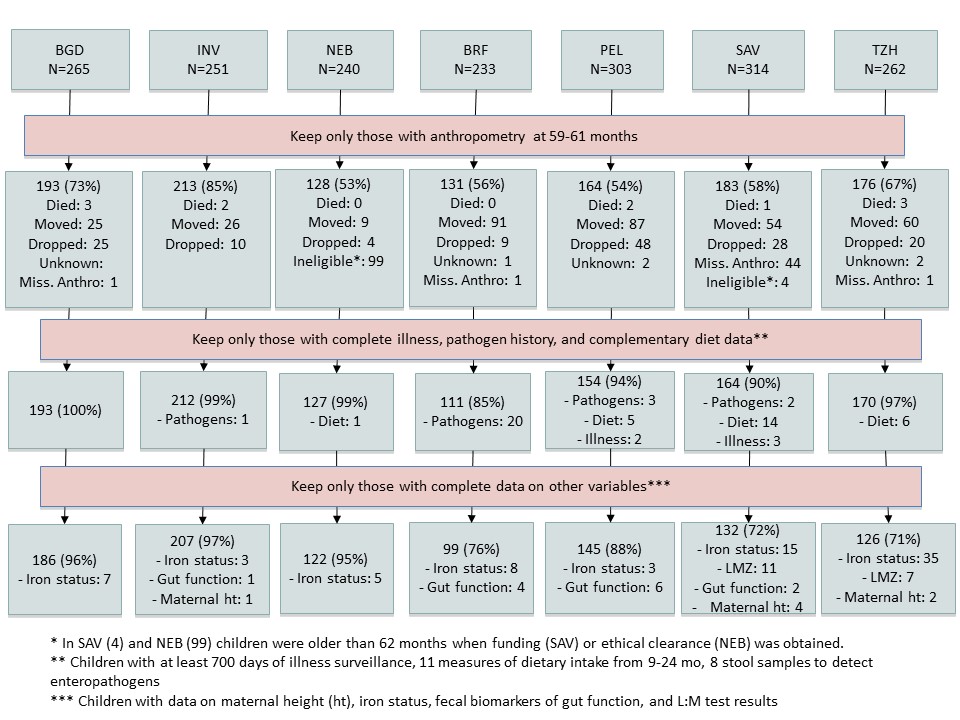


Supplemental Figure 2. Coefficients for early life influences on height at five years of age (height for age z-score) overall and by site, with TAQ bacterial data [Sites: BGD: Bangladesh – Dhaka; INV: India – Vellore; NEB: Nepal – Bhaktapur; BRF: Brazil – Fortaleza; PEL: Peru – Loreto; SAV: South Africa – Venda; TZH: Tanzania – Haydom]


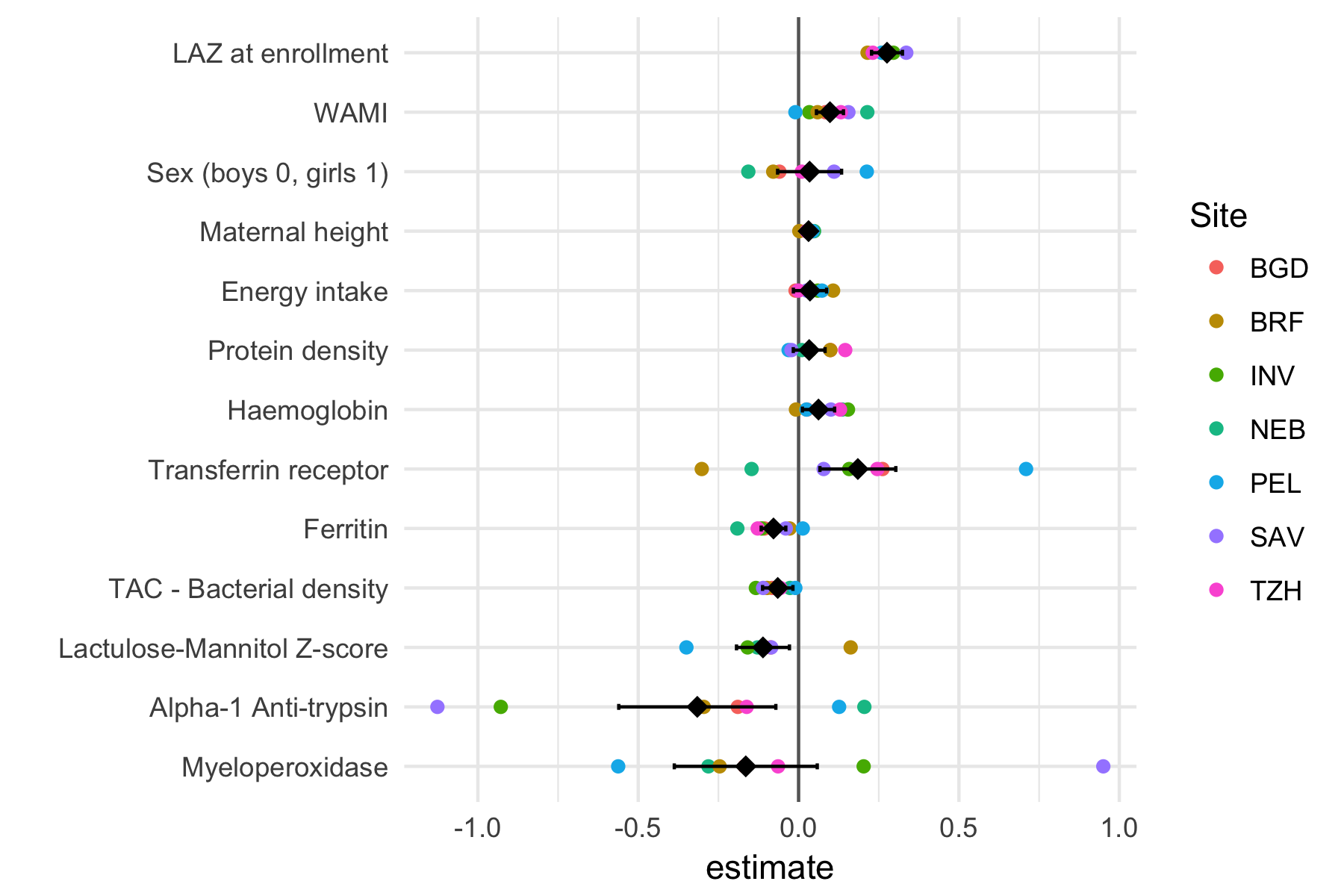


Supplemental Figure 3. Coefficients for early life influences on weight at five years of age (weight for age z-score) overall and by site [Sites: BGD: Bangladesh – Dhaka; INV: India – Vellore; NEB: Nepal – Bhaktapur; BRF: Brazil – Fortaleza; PEL: Peru – Loreto; SAV: South Africa – Venda; TZH: Tanzania – Haydom]


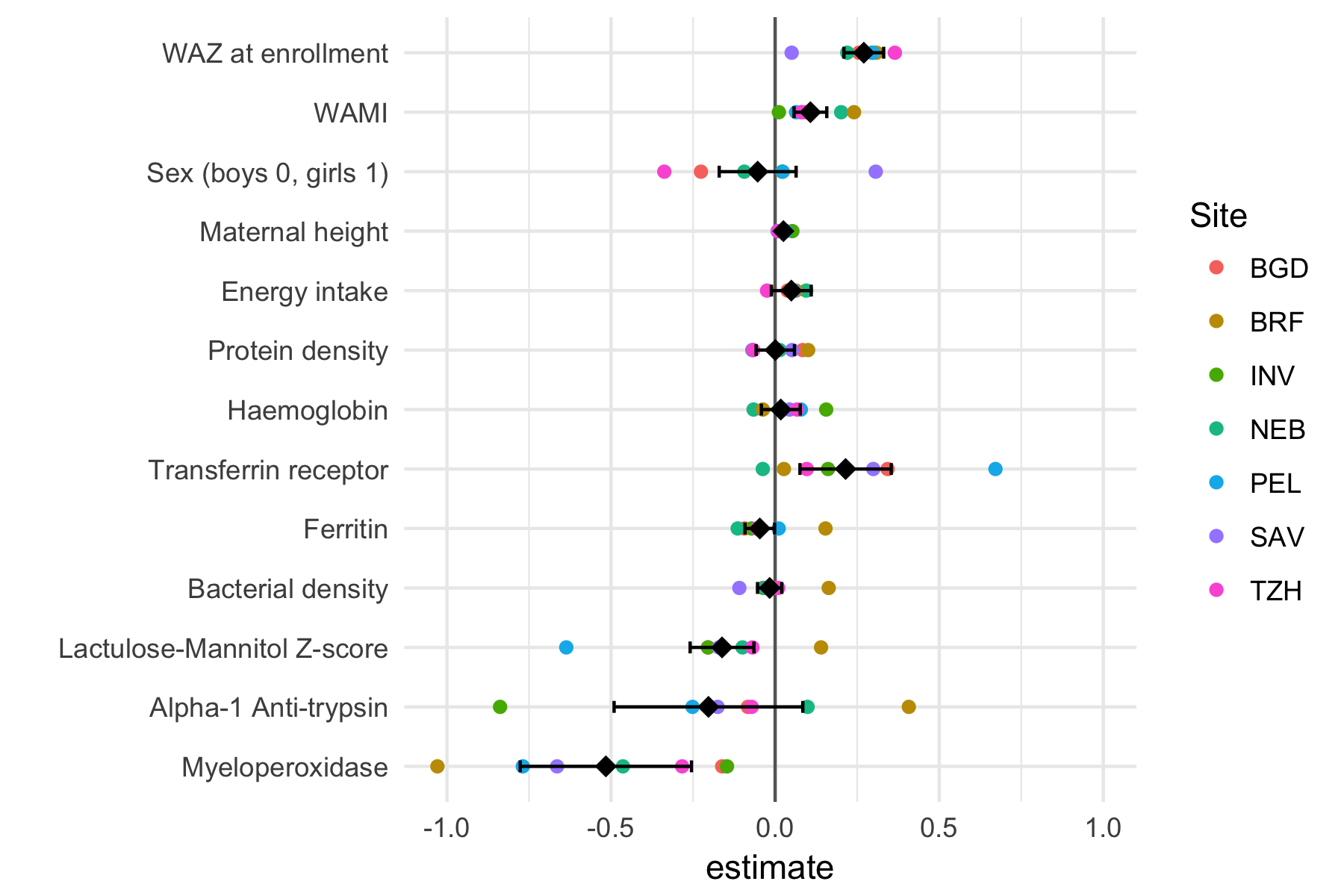


Supplemental Figure 4. Coefficients for early life influences on BMIZ at five years of age overall and by site [Sites: BGD: Bangladesh – Dhaka; INV: India – Vellore; NEB: Nepal – Bhaktapur; BRF: Brazil – Fortaleza; PEL: Peru – Loreto; SAV: South Africa – Venda; TZH: Tanzania – Haydom]


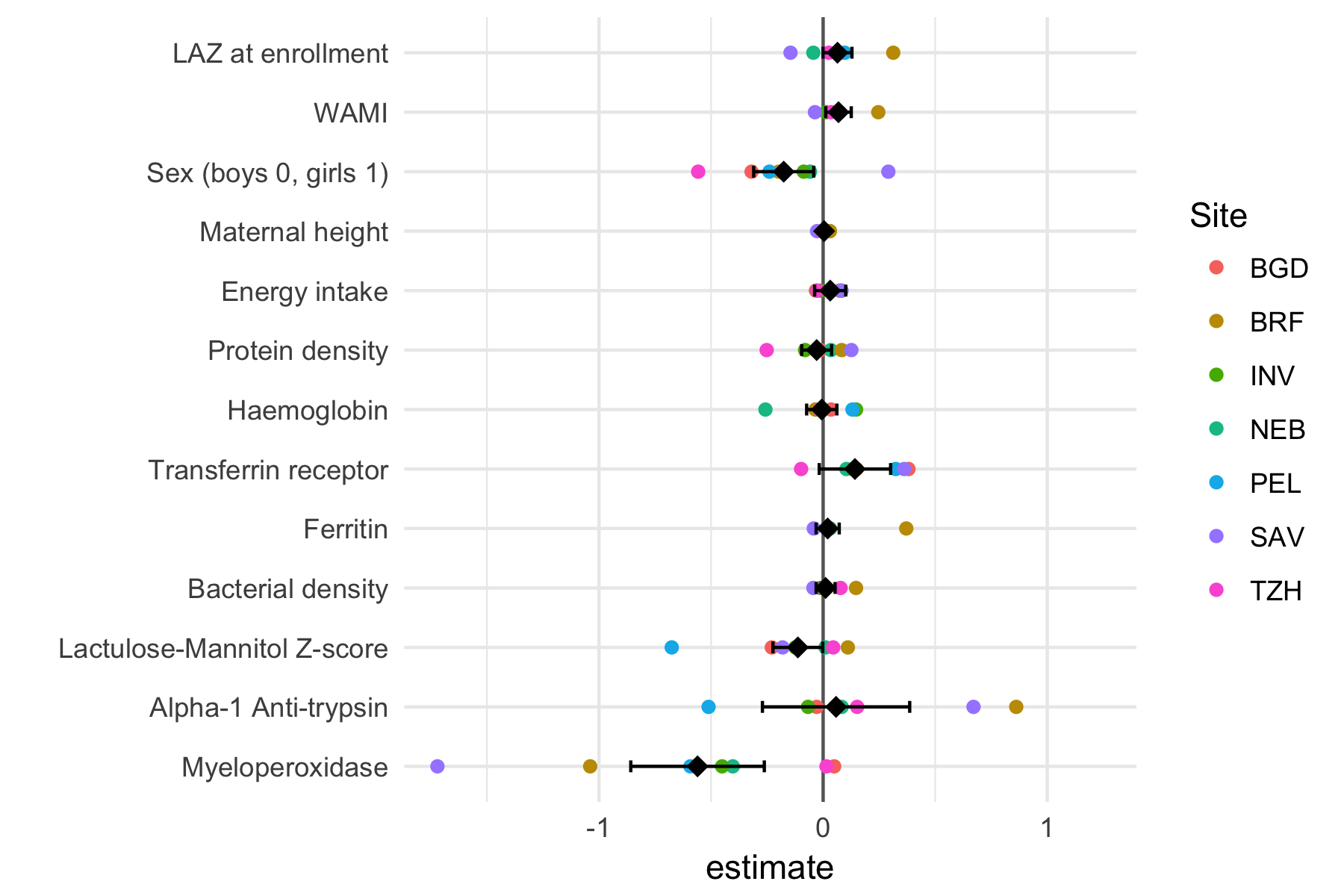

Supplement: nqz004_Supplemental_File [file nqz004_supplemental_file.docx]
